# Multi-Class Arrhythmia Detection from PPG Signals Based on VGG-BiLSTM Hybrid Deep Learning Model

**DOI:** 10.3390/bios16050235

**Published:** 2026-04-23

**Authors:** Shiyong Li, Jiaying Mo, Jiating Pan, Zhengguang Zheng, Qunfeng Tang, Zhencheng Chen

**Affiliations:** 1 School of Electronic Engineering and Automation, Guilin University of Electronic Technology, Guilin 541004, China; lishiyong@guet.edu.cn (S.L.); mojiaying@mails.guet.edu.cn (J.M.); 21082201015@mails.guet.edu.cn (J.P.); 2 School of Life and Environmental Sciences, Guilin University of Electronic Technology, Guilin 541004, China

**Keywords:** arrhythmia classification, photoplethysmography, VGG-BiLSTM, multi-class classification

## Abstract

Arrhythmia is a common and potentially life-threatening cardiovascular condition. Photoplethysmography (PPG) has emerged as a noninvasive alternative to electrocardiography for cardiac rhythm monitoring, yet most PPG-based methods remain limited to binary classification. In this study, a new deep learning approach is suggested for categorizing six arrhythmia types from PPG data: sinus rhythm (SR), premature ventricular contraction (PVC), premature atrial contraction (PAC), ventricular tachycardia (VT), supraventricular tachycardia (SVT), and atrial fibrillation (AF). The raw PPG signal is enhanced by extracting its first and second derivatives to capture morphological features not readily apparent in the original signal. A hybrid architecture, VGG-BiLSTM, is utilized, merging VGG convolutional layers for spatial features extraction with bidirectional long short-term memory layers for modeling temporal dependencies. A stratified data splitting strategy is further adopted to address class imbalance across arrhythmia types. A publicly available dataset containing 46,827 PPG segments from 91 individuals was employed to assess the effectiveness of the suggested technique. The method yielded an overall accuracy, sensitivity, specificity and F1 score of 88.7%, 78.5%, 97.6% and 80.5% correspondingly.

## 1. Introduction

Cardiovascular disease (CVD) is the primary global cause of mortality [1], with arrhythmia as a prevalent cardiovascular condition on the rise in recent years. The World Health Organization reports approximately 17.9 million CVD-related deaths annually [2]. Arrhythmia is mainly characterized by abnormalities in the frequency and rhythm of the heartbeat [3,4]. Numerous cardiac rhythm abnormalities exist, including PVC, PAC, VT, SVT, and AF [5,6]. Minor arrhythmias can result in discomfort like chest tightness and weakness, affecting daily activities. On the other hand, severe arrhythmias can cause substantial cardiac function impairment or heart failure, and in extreme cases, sudden death [7,8]. Therefore, how to effectively prevent and diagnose arrhythmias has become a major challenge. Timely identification of arrhythmias can help physicians provide more precise treatment and greatly reduce the risk of mortality. Currently, the electrocardiogram (ECG) is the most common noninvasive and cost-effective tool for diagnosing arrhythmias and is widely regarded as the gold standard for arrhythmia detection [9,10,11,12]. Although ECG monitoring has a high diagnostic accuracy, it still has some limitations in practical applications. For example, during prolonged examinations, electrodes must be placed on the patient’s skin, which may cause irritation, allergic reactions, or discomfort. Furthermore, the required multi-lead setup may restrict the patient’s activities, interfere with their daily life, and cause considerable inconvenience.

In recent years, PPG has gained significant traction in medical research for monitoring cardiac activity and vascular health due to its affordability, user-friendliness, and noninvasiveness [13]. The PPG signal is an optically obtained measurement of changes in blood volume during the cardiac cycle, acquired from peripheral body sites such as the fingers, wrists, and forehead [14]. Essentially, it represents a pulse-pressure waveform stemming from cardiac contraction and traveling through the vascular system. Optical volumetric pulse wave technology has the advantage of easy signal acquisition, portability, and wearability due to its small sensor size, which means that patients can wear this device for continuous monitoring for long periods of time without much disruption to their daily lives. Additionally, given the strong physiological correlation between PPG and ECG signals, PPG can function as a feasible substitute for detecting arrhythmias, particularly in portable, noninvasive monitoring setups [15].

Researchers have developed various arrhythmia classification models based on PPG signals using artificial intelligence advancements. These models are categorized into machine learning (ML) and deep learning (DL). ML methods such as artificial neural network (ANN) [16,17], random forest (RF) [18,19], support vector machine [20,21,22], and decision tree (DT) [23,24]. However, ML methods depend on manual feature extraction, which requires substantial domain expertise and risks omitting important signal information, potentially causing misdiagnosis. DL has emerged as a powerful tool for medical signal processing because DL models learn feature representations automatically, eliminating the tedious task of feature engineering. However, most existing research focuses on binary classification tasks, especially the classification of AF and SR, and usually achieves high performance metrics. For example, McManus et al. [25] used statistical features including RMSSD/SbE to detect atrial fibrillation from PPG signals, achieving a sensitivity of 96.2% and a specificity of 97.5%. Bashar et al. [26] further improved the performance of binary atrial fibrillation detection by integrating RMSSD, SampleEn, and PAC/PVC detection, achieving a sensitivity of 98.18% and a specificity of 97.43%. Cheng et al. [27] combined time–frequency analysis with deep learning to propose a hybrid model that integrates a 2D convolutional neural network and a long short-term memory network. They converted PPG signals into time–frequency spectrograms using continuous wavelet transform and achieved 98.00% sensitivity, 98.07% specificity, and an F1 score of 98.13% for AF versus SR classification. Although these binary classification methods perform well, their clinical application has inherent limitations because actual cardiac monitoring requires the simultaneous identification of multiple arrhythmia types. Because different heart rhythm types are similar in morphology, detecting multiple arrhythmias from PPG signals faces greater challenges. Liu et al. [28] made up for this deficiency by constructing a six-class arrhythmia classification framework. The framework was tested on a self-built dataset containing 118,217 PPG samples from 228 patients using a deep convolutional neural network (DCNN), and finally achieved an accuracy of 85.0%, a sensitivity of 75.8%, and an F1 score of 75.2%. However, their model had limitations in distinguishing morphologically similar arrhythmias, such as ventricular premature beats versus atrial premature beats, and ventricular tachycardia versus supraventricular tachycardia. This finding suggests that pure convolutional methods may not be able to fully utilize the inherent time dependence in PPG signals for fine-grained arrhythmia identification.

To address the limitations described above, this study proposes a hybrid deep-learning architecture based on VGG and BiLSTM for multi-class arrhythmia classification from PPG signals. The main contributions and innovations are as follows:

Differential feature enhancement for PPG signals: Unlike conventional methods that use raw PPG signals directly, the proposed framework integrates derivative-based feature augmentation. Specifically, first-order and second-order differentiation operations are applied to the original PPG signals to generate velocity plethysmography (VPG) and acceleration plethysmography (APG), respectively. This processing constructs a more comprehensive feature representation space and enables the capture of subtle morphological variations that are indistinguishable in raw PPG signals.

Hierarchical data partitioning for class-imbalance mitigation: A hierarchical data-splitting strategy is adopted to ensure proportional distribution of each arrhythmia category across the training, validation, and test sets. This approach effectively alleviates the inherent class-imbalance problem in arrhythmia datasets and reduces model bias toward majority classes.

Spatio-temporal feature fusion via VGG-BiLSTM architecture: The proposed VGG-BiLSTM framework combines the spatial feature-extraction strength of VGG convolutional layers with the bidirectional temporal modeling capability of BiLSTM layers. This integration facilitates a comprehensive characterization of the contextual features of cardiac rhythm patterns.

## 2. Materials and Methods

### 2.1. Dataset Description

This study used the PPG signal dataset published by Liu et al. [28], which contains complete data from 91 patients with arrhythmias who underwent radiofrequency ablation. This dataset accounts for 40% of the original complete dataset from Liu et al., but it is not an arbitrarily screened or cleaned subset—instead, it is the only complete publicly available portion of the original dataset, as the full dataset involves clinical privacy data from medical centers and is not fully open-sourced due to ethical and privacy protection requirements. It includes all validation and test sets used in the original study, maintaining the same data standards as the baseline experiment. The dataset contains 46,827 PPG signal segments, each lasting 10 s, with a sampling frequency of 100 Hz, and each segment contains 1000 discrete data points.

**Figure 1:**
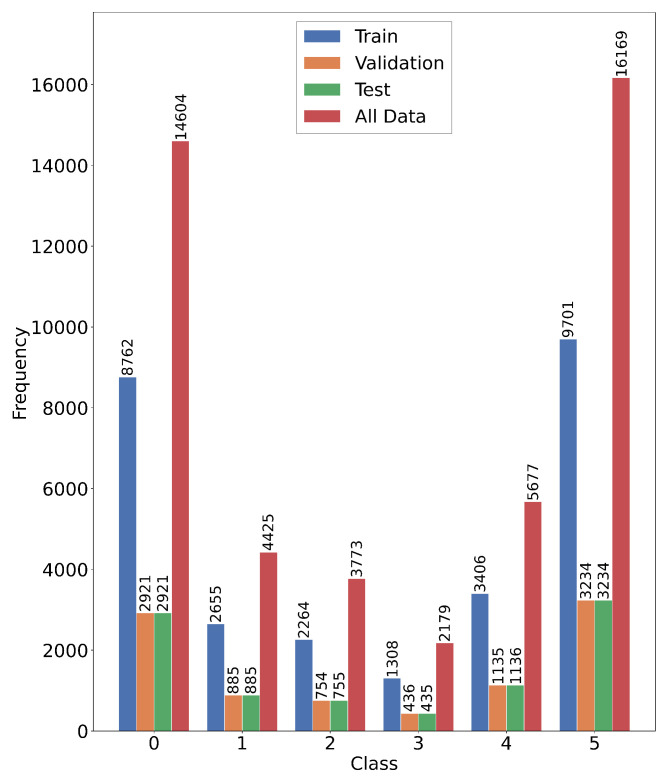
Distribution of PPG signal samples across six arrhythmia categories in the dataset.

As shown in Figure 1, the dataset comprises SR as the normal heart rhythm category, along with PVC, PAC, VT, SVT, and AF. Each rhythm type is assigned a numerical label from 0 to 5. The distribution of samples from different rhythm types shows a significant class imbalance, with atrial fibrillation accounting for the highest proportion at 34.5% (16,169 samples), while ventricular tachycardia accounts for the lowest proportion at 4.7% (2179 samples). This imbalance poses challenges for model training and evaluation, and thus requires special handling strategies, which will be described in the subsequent sections.

### 2.2. Multi-View Signal Processing Framework

#### 2.2.1. Derivative-Based Feature Enhancement

The current literature uses PPG signals to detect arrhythmias, but we incorporate velocity-volume pulse wave and acceleration-volume pulse wave signals to provide more feature representations. VPG is the first derivative of PPG signal, representing instantaneous change in blood flow velocity and providing information such as change rate of peripheral vascular volume. APG is the second derivative of PPG signal and reflects dynamic change in blood flow acceleration. Previous studies show that APG signal is related to arterial aging and stiffness [29].

In this study, the derivative calculation is implemented using the first-order backward finite difference method, combined with a symmetric boundary extrapolation strategy to ensure the consistency of signal length. For all sampling points *i* > 0, the backward difference is adopted to strictly approximate the instantaneous derivative, which only uses historical and current samples and satisfies the causality requirement of real-time physiological signal processing. For the first element (i = 0) of each sequence, where no previous sampling point exists, we perform symmetric boundary extrapolation based on the backward difference of the initial two points, ensuring that the derivative signal has the same dimension as the original PPG signal while maintaining waveform continuity. The mathematical formula for VPG calculation is as follows:

(1) $$\mathrm{VPG}\left[i\right]=\left\{\begin{array}{cc} \mathrm{PPG}[0]-(\mathrm{PPG}[1]-\mathrm{PPG}[0]), & i=0\\ \mathrm{PPG}[i]-\mathrm{PPG}[i-1], & i>0 \end{array}\right.$$

Similarly, the APG signal, as the second derivative of the PPG signal, is computed by applying the same backward finite difference scheme to the VPG signal:

(2) $$\mathrm{APG}\left[i\right]=\left\{\begin{array}{cc} \mathrm{VPG}[0]-(\mathrm{VPG}[1]-\mathrm{VPG}[0]), & i=0\\ \mathrm{VPG}[i]-\mathrm{VPG}[i-1], & i>0 \end{array}\right.$$

This derivative-based feature extraction method can capture subtle morphological changes in PPG signals that are difficult to detect in the original waveform. It is particularly valuable for differentiating arrhythmias with similar morphology, such as ventricular premature beats and atrial premature beats, which often pose classification challenges due to their overlapping waveform characteristics and temporal patterns.

#### 2.2.2. Signal Normalization

In order to minimize the physiological differences among individuals and compensate for the common baseline drift artifacts in the photoplethysmography (PPG) recordings, each 10-s PPG segment was normalized by Z-score method segment by segment. The specific formula is as follows:

(3) $${\mathrm{PPG}}_{\mathrm{norm}}=\frac{\mathrm{PPG}-\mu }{\sigma }$$

where μ represents the piecewise mean, and σ represents the standard deviation of 1000 data points within each segment. This normalization strategy retains the relative amplitude changes within each segment, ensures the numerical stability of the entire dataset, and is conducive to more efficient gradient propagation during the training process of neural networks.

### 2.3. Stratified Data Splitting Strategy

When handling datasets with imbalanced class distributions, traditional random partitioning often produces uneven class proportions across training, validation, and test sets. This imbalance causes biased model evaluation and limits generalization performance, especially for minority classes. To address this problem, this study adopted a stratified sampling approach to ensure fair representation of each class within all data partitions. The dataset was partitioned into training, validation, and test sets at a ratio of 60:20:20 while strictly maintaining the stratified constraint. Specifically, the training set contained 28,097 samples, and the validation and test sets each contained 9365 samples.

The stratified partitioning proceeded in two stages. In the first stage, the entire dataset was split into a training set and a temporary set at a ratio of 60:40 while preserving class balance. In the second stage, the temporary set was equally divided into validation and test sets, again preserving class proportions. The detailed procedure of this method is described as follows:

(4) $$D_{\mathrm{train}},D_{\mathrm{temp}}=\mathrm{StratifiedSplit}\left(D,y,\mathrm{train}\_\mathrm{size}=0.6,\;\mathrm{stratify}=y\right)$$

(5) $$D_{\mathrm{val}},D_{\mathrm{test}}=\mathrm{StratifiedSplit}\left(D_{\mathrm{temp}},y_{\mathrm{temp}},\mathrm{test}\_\mathrm{size}=0.5,\;\mathrm{stratify}=y_{\mathrm{temp}}\right)$$

To ensure the reproducibility of the experimental results, the random seeds for all experiments were fixed at 42. Given the severe class imbalance problem in the dataset, this stratified method is particularly important in the model training process. This approach prevents bias towards the majority class, ensuring an unbiased performance evaluation across all rhythm types.

### 2.4. Deep Learning Architectures

#### 2.4.1. Baseline VGG Architecture

The baseline model uses a VGG architecture adapted for one-dimensional PPG signal processing [30]. The input consists of PPG, VPG, and APG signals stacked along the channel dimension to form a three-channel input tensor. This architecture consists of three convolutional blocks with increasing filter size to extract features at multiple time scales. Each convolutional block consists of two convolutional layers, Batch Normalization and ReLU activation ending with max pooling.

The first convolutional block transforms the input signal from a single channel to 64 feature maps through 3-kernel convolutions with padding to preserve temporal dimensions. Following max pooling with stride 2, the temporal dimension reduces from 1000 to 500 points. The second block expands the feature space to 128 channels while further reducing temporal dimension to 250 points. The third block achieves maximum feature extraction with 256 channels and 125 temporal points.

The extracted convolutional features undergo flattening to create a 32,000-dimensional feature vector (256 × 125), which is subsequently processed through a three-layer fully connected classifier. The classifier architecture incorporates two hidden layers with 4096 neurons each, utilizing ReLU activation and 50% dropout for regularization. The final layer performs six-class classification through softmax activation.

#### 2.4.2. VGG-BiLSTM Hybrid Architecture

**Figure 2:**
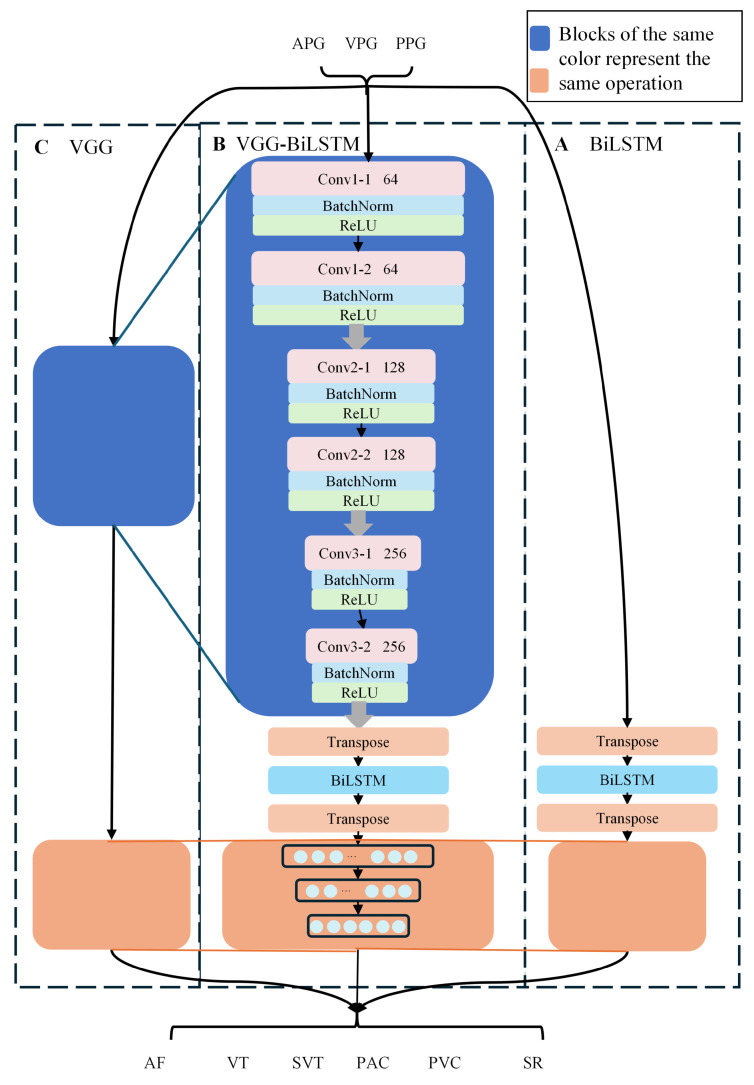
Architecture of the proposed VGG-BiLSTM model.

The proposed VGG-BiLSTM architecture extends the baseline model by incorporating bidirectional long short-term memory layers to capture temporal dependencies inherent in PPG signals [31,32]. Following the third convolutional block, the feature maps undergo transposition from (batch_size, channels, sequence_length) to (batch_size, sequence_length, channels) format to conform with LSTM input requirements. The complete architecture is illustrated in Figure 2.

The BiLSTM module consists of two stacked bidirectional LSTM layers, each with 128 hidden units per direction, resulting in a combined output dimension of 256 features. The bidirectional processing enables the model to capture both forward and backward temporal dependencies, crucial for understanding the complete context of cardiac rhythm patterns. The mathematical formulation of bidirectional processing is expressed as:

(6) $${\vec{h}}_{t}={\mathrm{LSTM}}_{\mathrm{forward}}\left(x_{t},{\vec{h}}_{t-1},{\vec{c}}_{t-1}\right)$$

(7) $${\overset{\leftarrow }{h}}_{t}={\mathrm{LSTM}}_{\mathrm{backward}}\left(x_{t},{\overset{\leftarrow }{h}}_{t+1},{\overset{\leftarrow }{c}}_{t+1}\right)$$

(8) $$h_{t}=\left[{\vec{h}}_{t};{\overset{\leftarrow }{h}}_{t}\right]$$

where ${\vec{h}}_{t}$ and ${\overset{\leftarrow }{h}}_{t}$ represent forward and backward hidden states respectively, while ${\vec{c}}_{t}$ and ${\overset{\leftarrow }{c}}_{t}$ denote the corresponding cell states. The concatenated bidirectional features preserve the sequence length of 125 time steps, resulting in a flattened dimension of 32,000 (256 × 125) before entering the fully connected classifier.

### 2.5. Training Configuration and Optimization

Model training used the Adam optimizer, which adaptively adjusted the learning rate by estimating the first and second moments of the gradient. Training parameters for both architectures were kept consistent: the initial learning rate was $10^{-4}$ and the batch size was 32 samples. This configuration balanced computational efficiency and gradient stability to meet the model training requirements. The maximum number of training iterations was 200 epochs; to prevent overfitting and preserve convergence, an early stopping strategy was adopted, and training was terminated if validation loss showed no improvement for 20 consecutive epochs. In this study, the cross-entropy loss function was chosen to compute the training loss, and its specific calculation method is as follows:

(9) $$L=-\frac{1}{N}\sum_{i=1}^{N}\sum_{j=1}^{C}y_{ij}\log\left({\hat{y}}_{ij}\right)$$

Here, *N* represents the batch size, *C* represents the number of categories (6), $y_{ij}$ represents the true label, and ${\hat{y}}_{ij}$ represents the predicted probability that sample *i* belongs to category *j*.

Regularization techniques include applying a dropout rate of 0.5 to the fully connected layers and performing batch normalization after each convolutional layer. The application of these methods can both prevent overfitting and maintain the model’s expressive power.

### 2.6. Evaluation Metrics

The model performance is assessed by a set of indicators. The precision measures the number of correct predictions of positive samples; the sensitivity measures the number of correct predictions of negative samples. The specificity measures the number of correct predictions. The F1 score measures the total number of correct predictions of the test set. The overall accuracy measures the number of correct predictions.

For each category *i*, the calculation methods of these indicators are as follows:

(10) $${\mathrm{Precision}}_{i}=\frac{TP_{i}}{TP_{i}+FP_{i}}\times 100\%$$

(11) $${\mathrm{Sensitivity}}_{i}=\frac{TP_{i}}{TP_{i}+FN_{i}}\times 100\%$$

(12) $${\mathrm{Specificity}}_{i}=\frac{TN_{i}}{TN_{i}+FP_{i}}\times 100\%$$

(13) $$F1\;{\mathrm{Score}}_{i}=2\times \frac{{\mathrm{Precision}}_{i}\times {\mathrm{Sensitivity}}_{i}}{{\mathrm{Precision}}_{i}+{\mathrm{Sensitivity}}_{i}}\times 100\%$$

where $TP_{i}$, $TN_{i}$, $FP_{i}$, and $FN_{i}$ represent true positives, true negatives, false positives, and false negatives for class *i* respectively.

The overall accuracy is computed as:

(14) $$\mathrm{Overall}\;\mathrm{Accuracy}=\frac{\sum_{i=1}^{C}TP_{i}}{\sum_{i=1}^{C}\left(TP_{i}+FP_{i}+TN_{i}+FN_{i}\right)}\times 100\%=\frac{\sum_{i=1}^{C}TP_{i}}{N}\times 100\%$$

where *C* is the total number of classes and *N* is the total number of test samples.

### 2.7. Implementation Environment

All experiments were implemented using the PyTorch 1.12.1 deep learning framework. Model training and evaluation were conducted on a workstation equipped with an NVIDIA GeForce RTX 2080 Ti GPU (11 GB of memory), an Intel Core i7-12700KF CPU, and 64 GB of system memory. The operating system was Windows 11. Throughout the entire experiment, fixed random seeds and deterministic operations were adopted to ensure the reproducibility of the results.

## 3. Results

### 3.1. Comparative Experimental Results

Table 1 presents a complete ablation study comparing four progressive configurations to quantify the individual contribution of each proposed component:

- BiLSTM—to evaluate the standalone temporal modeling capability without convolutional feature extraction;
- VGG—to evaluate the standalone spatial feature extraction capability;
- VGG-BiLSTM (PPG only) — to evaluate the hybrid architecture using only the raw PPG signal as input, thereby isolating the contribution of the derivative enhancement;
- Our Method—the full model with both the hybrid architecture and derivative-enhanced multi-signal input.

**Table 1:** Ablation study: detailed performance comparison of different model configurations on six-class arrhythmia classification task. Values are presented as mean (95% CI).

Model	Rhythm Type	Precision (%)	Sensitivity (%)	Specificity (%)	F1 Score (%)	Accuracy (%)
BiLSTM	SR	94.1 (93.2–94.9)	98.3 (97.8–98.7)	97.2 (96.8–97.6)	96.1 (95.4–96.7)	80.5 (79.7–81.3)
	PVC	69.4 (65.4–73.1)	42.5 (39.3–45.8)	98.0 (97.7–98.3)	52.7 (49.1–56.3)	
	PAC	52.7 (47.2–58.1)	22.1 (19.3–25.2)	98.3 (98.0–98.5)	31.2 (27.4–35.2)	
	VT	67.9 (62.4–72.9)	47.1 (42.5–51.8)	98.9 (98.7–99.1)	55.6 (50.6–60.6)	
	SVT	79.9 (77.5–82.1)	79.5 (77.0–81.7)	97.2 (96.9–97.6)	79.7 (77.3–81.9)	
	AF	75.0 (73.7–76.4)	93.4 (92.4–94.2)	83.6 (82.7–84.5)	83.2 (82.0–84.3)	
	Mean	73.2 (62.1–84.2)	63.8 (39.1–88.5)	95.5 (90.8–100.0)	66.4 (47.2–85.7)	
VGG	SR	94.8 (93.9–95.5)	97.5 (96.8–98.0)	97.6 (97.2–97.9)	96.1 (95.4–96.7)	85.4 (84.7–86.1)
	PVC	67.6 (64.4–70.6)	65.9 (62.7–68.9)	96.7 (96.3–97.1)	66.7 (63.5–69.7)	
	PAC	66.0 (61.8–69.9)	44.9 (41.4–48.5)	98.0 (97.6–98.2)	53.4 (49.6–57.2)	
	VT	71.1 (66.5–75.3)	66.7 (62.1–70.9)	98.7 (98.4–98.9)	68.8 (64.2–73.0)	
	SVT	82.0 (79.7–84.1)	81.9 (79.5–84.0)	97.5 (97.2–97.8)	81.9 (79.6–84.1)	
	AF	87.4 (86.2–88.5)	93.0 (92.1–93.9)	92.9 (92.3–93.5)	90.1 (89.1–91.1)	
	Mean	78.1 (68.7–87.5)	75.0 (59.2–90.7)	96.9 (95.3–98.5)	76.2 (63.3–89.0)	
VGG-BiLSTM (PPG only)	SR	96.7 (96.0–97.3)	98.9 (98.4–99.2)	98.5 (98.2–98.8)	97.8 (97.2–98.2)	88.0 (87.4–88.7)
	PVC	80.9 (78.0–83.6)	69.0 (65.9–72.0)	98.3 (98.0–98.6)	74.5 (71.4–77.4)	
	PAC	72.6 (68.9–76.0)	56.8 (53.3–60.3)	98.1 (97.8–98.4)	63.7 (60.1–67.3)	
	VT	72.6 (67.9–76.9)	63.4 (58.8–67.8)	98.8 (98.6–99.0)	67.7 (63.1–72.1)	
	SVT	88.9 (86.9–90.7)	78.6 (76.1–80.9)	98.7 (98.4–98.9)	83.5 (81.1–85.5)	
	AF	86.3 (85.1–87.4)	97.4 (96.8–97.9)	91.8 (91.1–92.5)	91.5 (90.6–92.3)	
	Mean	83.0 (75.4–90.6)	77.4 (63.3–91.4)	97.4 (95.2–99.6)	79.8 (69.0–90.5)	
Our Method	SR	97.3 (96.6–97.8)	98.8 (98.4–99.2)	98.7 (98.4–99.0)	98.0 (97.5–98.5)	88.7 (88.1–89.3)
	PVC	79.5 (76.5–82.2)	69.3 (66.1–72.2)	98.1 (97.8–98.4)	74.0 (71.0–76.9)	
	PAC	73.3 (69.5–76.8)	55.2 (51.7–58.7)	98.2 (97.9–98.5)	63.0 (59.3–66.6)	
	VT	75.3 (70.7–79.4)	65.1 (60.5–69.4)	99.0 (98.7–99.1)	69.8 (65.2–74.0)	
	SVT	86.0 (83.9–87.9)	85.1 (82.9–87.1)	98.1 (97.8–98.4)	85.6 (83.4–87.5)	
	AF	88.3 (87.2–89.3)	97.2 (96.6–97.7)	93.2 (92.6–93.8)	92.6 (91.7–93.3)	
	Mean	83.3 (76.1–90.5)	78.5 (64.1–92.8)	97.6 (95.8–99.3)	80.5 (69.5–91.5)	

All configurations are evaluated using five key performance indicators and their 95% confidence intervals: precision, sensitivity, specificity, F1 score, and overall accuracy.

The ablation results demonstrate progressive performance gains as each component is introduced. The standalone BiLSTM model achieved the lowest overall accuracy of 80.5%, with notably poor sensitivity for PAC at 22.1% and PVC at 42.5%, indicating that purely temporal modeling is insufficient to capture the morphological differences required for ectopic beat detection. The VGG model improved the overall accuracy to 85.4%, an increase of 4.9 percentage points, and the confidence intervals of these two models do not overlap, confirming statistical significance. The VGG-BiLSTM hybrid architecture with PPG-only input further elevated the overall accuracy to 88.0%, an additional increase of 2.6 percentage points over the VGG model, again with non-overlapping confidence intervals. These results validate that bidirectional temporal modeling effectively complements the spatial features learned by the convolutional layers.

The full proposed method with derivative-enhanced input achieved the highest overall accuracy of 88.7%, representing an improvement of 0.7 percentage points over the PPG-only variant. While the overall accuracy gain is modest, the derivative enhancement produced significant class-specific improvements. The sensitivity of SVT increased from 78.6% to 85.1%, an increase of 6.5 percentage points, and the confidence intervals of these two configurations do not overlap, confirming the statistical significance of the derivative contribution. The specificity of AF also improved from 91.8% to 93.2% with non-overlapping confidence intervals, indicating that the first- and second-order derivatives of the PPG signal provide important morphological information for reducing false positive detections.

The confidence intervals of the overall performance indicators also indicate an improvement in statistical reliability. The confidence intervals of the overall accuracy rates of the VGG model and the VGG-BiLSTM model do not overlap. The results of our model have high specificity across all categories, which is crucial for clinical applications and can minimize false positive predictions that lead to unnecessary interventions.

### 3.2. Confusion Matrix Analysis

**Figure 3:**
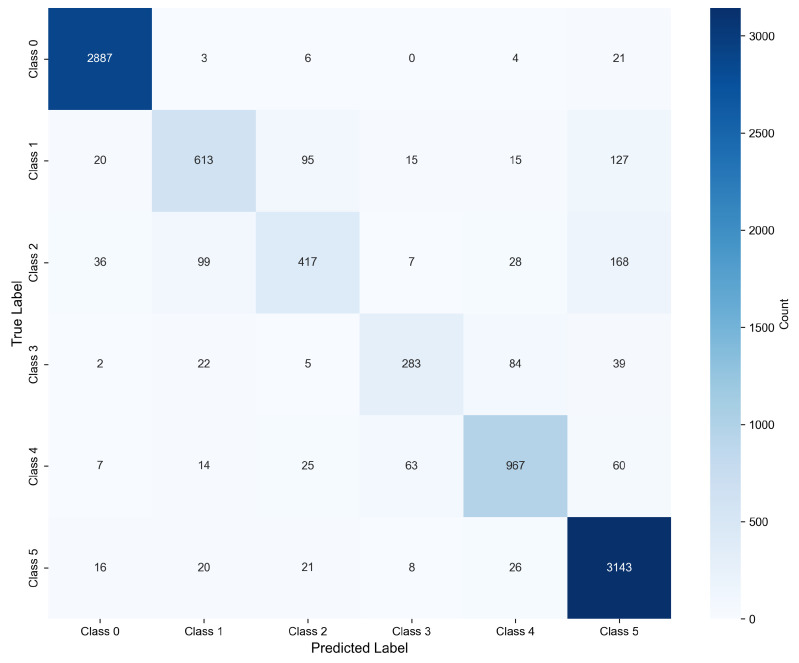
Confusion matrix of VGG-BiLSTM model, where class 0–5 represent SR, PVC, PAC, VT, SVT, and AF respectively.

Figure 3 presents the confusion matrix of the VGG-BiLSTM model, which reveals the detailed classification error distribution for different rhythm categories.

The results of the confusion matrix provide preliminary evidence for the classification performance of our model in sinus rhythm (SR) and atrial fibrillation (AF). The model correctly classified 2887 out of 2921 SR samples, achieving a correct classification rate of 98.8%, with only a few samples being misclassified into other categories. The classification performance for AF was also satisfactory, with 3143 out of 3234 samples being correctly identified, achieving a correct classification rate of 97.2%. These results suggest that the model has the potential to distinguish between normal heart rhythm and persistent arrhythmias associated with AF, though further verification is needed to confirm its clinical applicability. The main challenge in multi-classification problems lies in the similarities of physiological features among heart rhythms. There is a significant cross-misclassification between ventricular premature beats (PVC) and atrial premature beats (PAC), with correct classification rates of 69.3% and 55.2% respectively. A possible reason for this cross-classification is the similar morphological characteristics of ectopic beats, which requires further feature analysis to verify. Similarly, ventricular tachycardia (VT) and supraventricular tachycardia (SVT) also show obvious confusion, with 19.3% of VT samples being wrongly classified as SVT. Clinically, both are rapid and persistent arrhythmias, which may lead to the similarity of their characteristic signals captured by the model, but this inference needs to be supported by more detailed feature analysis.

### 3.3. Feature Space Visualization

**Figure 4:**
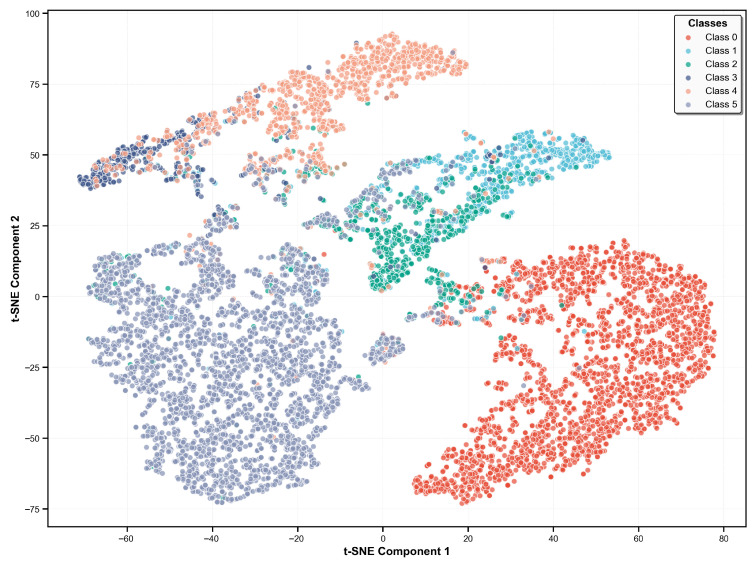
t-SNE visualization of learned feature representations from VGG-BiLSTM model, where class 0–5 represent SR, PVC, PAC, VT, SVT, and AF respectively.

Figure 4 presents intuitive auxiliary information for the model’s classification performance by showing the clustering patterns of different heart rhythm samples. Specifically, the SR group forms a well-defined and compact cluster, which is consistent with its high classification sensitivity of 98.8%. In contrast, the AF group occupies an independent region, which is in line with its 97.2% sensitivity, suggesting that the model has a certain ability to distinguish between regular and irregular rhythms. For ectopic beats (including PVC and PAC), a significant overlap is observed in the central region of the t-SNE plot, which is consistent with the mutual confusion between these two categories shown in the confusion matrix, indicating that their feature differences are not sufficient for the model to achieve accurate classification. Similarly, partial overlap is found between the VT and SVT groups, which may be related to the similar signal characteristics of the two arrhythmias.

### 3.4. Comparison with Existing PPG-Based Arrhythmia Detection Methods

To summarize the performance of our proposed VGG-BiLSTM model, we compared it with existing PPG-based arrhythmia detection methods from literature. Table 2 summarizes classification performance of different studies, including dataset size, target classification, methods, and results.

Comparative analysis indicates that performance varies with classification complexity. Studies focusing on binary classification of atrial fibrillation (AF) and sinus rhythm (SR) have all achieved excellent results. Cheng et al. reported the best performance, with a sensitivity of 98.00%, specificity of 98.07%, and F1-score of 98.13%. Binary classification tasks are simplified in nature, which reduces computational complexity and enables more focused feature learning, thereby improving accuracy.

In contrast, multi-class arrhythmia detection is more challenging due to the diverse types of cardiac rhythms. Liu et al. used a deep convolutional neural network on a large dataset including 228 patients and 118,217 samples, and achieved an accuracy of 85.0%, sensitivity of 75.8%, and F1-score of 75.2% in the six-class classification task.

Our VGG-BiLSTM model was tested on a subset of the original dataset, which contained 46,827 samples from 91 patients. The results show that the model achieved an overall accuracy of 88.7%, precision of 83.3%, sensitivity of 78.5%, F1-score of 80.5%, and specificity of 97.6%. Even though only about 40% of the original dataset was used, our model still showed competitive performance and outperformed the original model in some metrics. The higher F1-score (80.5% vs. 75.2%) and specificity (97.6% vs. 96.9%) prove that the VGG-BiLSTM hybrid architecture can better capture the spatial and temporal features of cardiac signals.

Notably, the overall classification accuracy of the method proposed in this study is 88.7%, with an average sensitivity of 78.5%, showing a certain discrepancy between the two metrics. This discrepancy is mainly due to the imbalanced sample distribution among different arrhythmia categories in the dataset: common arrhythmia classes with sufficient samples can achieve high sensitivity, while a few rare or borderline arrhythmia types have relatively low sensitivity due to limited training samples, thereby reducing the overall average sensitivity. In clinical arrhythmia classification scenarios, sensitivity is equally important as specificity, and its core significance lies in reflecting the model’s ability to correctly identify patients with actual arrhythmias and reduce false-negative results. False-negative results may lead to missed detection of malignant arrhythmias (such as ventricular arrhythmias and high-grade atrioventricular block), delaying clinical intervention and posing potential risks to patient safety. Based on the average sensitivity of 78.5% in this study, the model can effectively identify most arrhythmia events and can be used as an auxiliary tool for preliminary clinical arrhythmia screening to reduce the workload of clinicians in reading electrocardiograms. However, there is still room for optimization in the identification of rare and atypical arrhythmias, which needs further improvement to reduce the risk of missed diagnosis.

Compared with dedicated binary classifiers, our model achieved a sensitivity of 98.8% and an accuracy of 97.2%, maintaining good performance in binary classification. Distinguishing rhythms with similar morphology, such as ventricular tachycardia (VT) vs. supraventricular tachycardia (SVT) or premature ventricular contraction (PVC) vs. premature atrial contraction (PAC), is still a key challenge in multi-class arrhythmia detection.

## 4. Conclusions

Aiming at the technical limitations of existing multi-class arrhythmia classification methods, which mostly focus on binary classification and overly rely on a single convolutional feature extraction, this study proposes a hybrid VGG-BiLSTM deep learning architecture based on PPG signals for the accurate classification of multi-class arrhythmias. To further enhance the model performance, the architecture integrates three key designs: first, a derived feature enhancement strategy based on VPG and APG signals to enrich the signal feature dimensions; second, a hierarchical data partitioning method to effectively solve the class imbalance problem in the dataset; and third, the construction of a hybrid structure that fuses the VGG convolutional layer with the BiLSTM layer to fully utilize the advantages of spatial feature extraction of the VGG layer and the BiLSTM layer’s bidirectional time series modeling ability. In order to verify the effectiveness of the proposed model, experiments were carried out to evaluate the system on publicly available six types of arrhythmia datasets. The results show that this VGG-BiLSTM model has an overall accuracy of 88.7%, an average precision of 83.3%, an average sensitivity of 78.5%, an average F1 score of 80.5%, and an average specificity of 97.6%, which are superior to the baseline VGG architecture in all performance metrics. Compared with the DCNN model proposed by Liu et al. the model in this study only uses about 40% of the data volume of the original dataset but achieves better performance, in which the F1 score improves from 75.2% to 80.5% and specificity from 96.9% to 97.6%, confirming the superiority of the hybrid architecture in capturing the spatio-temporal joint features of PPG signals. Notably, the model excels in the detection of two arrhythmia types, sinus rhythm and atrial fibrillation, with sensitivities of 98.8% and 97.2%, respectively, and a performance level close to that of dedicated binary classifiers, which provides a feasible solution for the fast and efficient detection of multiple types of arrhythmias in the clinic.However, classification remains challenging for morphologically similar rhythm pairs, such as PVC versus PAC and VT versus SVT. The challenges faced by this multi-class problem stem from the inherent physiological similarities among these types of arrhythmias, as well as the limited discriminative information in the peripheral PPG signal. The main future research directions focus on enhancing the model’s ability to distinguish between morphologically similar types of arrhythmias; further exploring the contributions of different modules to the overall performance and the differentiation of similar arrhythmias and the mechanism of distinguishing a certain type of arrhythmia; and validating the model using dynamic PPG data collected from wearable devices in real-world environments to further evaluate its clinical application value.

## Figures and Tables

**Table 2:** Comparison between the proposed method and existing PPG-based arrhythmia detection methods.

Study	Dataset	Sample Size	Classification Target	No. of Classes	Method	Results
McManus et al. [25]	Self-constructed	76 subjects	AF vs. SR	2	RMSSD/mean + SbE	Sensitivity: 96.2% Specificity: 97.5% Precision: 96.8%
Bashar et al. [26]	UMass + Chonlab	46/366	AF vs. SR	2	RMSSD + SampEn +PAC/PVC detection	Sensitivity: 98.18% Specificity: 97.43% Precision: 97.54%
Cheng et al. [27]	MIMIC-III, etc.	102/28,440	AF vs. SR	2	2D-CNN + LSTM	Sensitivity: 98.00% Specificity: 98.07% Precision: 98.21% F1 Score: 98.13% AUC: 0.9959
Liu et al. [28]	Self-constructed	228/118,217	SR, PVC, PAC, VT, SVT, AF	6	DCNN	Sensitivity: 75.8% Specificity: 96.9% Precision: 85.0% F1 Score: 75.2% AUC: 0.978
This study	Liu et al. dataset	91/46,827	SR, PVC, PAC, VT, SVT, AF	6	VGG-BiLSTM	Precision: 83.3% Sensitivity: 78.5% Specificity: 97.6% F1 Score: 80.5% Accuracy: 88.7%

## Data Availability

The data used in this study were obtained from publicly available datasets by Liu et al. [28].
